# The Synthesis of Truncated Polypeptides for Immune Surveillance and Viral Evasion

**DOI:** 10.1371/journal.pone.0008692

**Published:** 2010-01-21

**Authors:** Sylvain Cardinaud, Shelley R. Starck, Piyanka Chandra, Nilabh Shastri

**Affiliations:** Division of Immunology and Pathogenesis, Department of Molecular and Cell Biology, University of California, Berkeley, California, United States of America; University of California San Francisco, United States of America

## Abstract

**Background:**

Cytotoxic T cells detect intracellular pathogens by surveying peptide loaded MHC class I molecules (pMHC I) on the cell surface. Effective immune surveillance also requires infected cells to present pMHC I promptly before viral progeny can escape. Rapid pMHC I presentation apparently occurs because infected cells can synthesize and present peptides from antigenic precursors called defective ribosomal products (DRiPs). The molecular characteristics of DRiPs are not known.

**Methodology/Principal Findings:**

Here, using a novel method for detecting antigenic precursors and proteolytic intermediates, we tracked the synthesis and processing of Epstein-Barr Virus encoded nuclear antigen 1 (EBNA1). We find that ribosomes initiated translation appropriately, but rapidly produced DRiPs representing ∼120 amino acid truncated EBNA1 polypeptides by premature termination. Moreover, specific sequences in EBNA1 mRNA strongly inhibited the generation of truncated DRiPs and pMHC I presentation.

**Significance:**

Our results reveal the first characterization of virus DRiPs as truncated translation products. Furthermore, production of EBNA1-derived DRiPs is down-regulated in cells, possibly limiting the antigenicity of EBNA1.

## Introduction

The MHC class I molecules present peptides (pMHC I) on the cell surface as potential targets for recognition by the CD8+ T cell repertoire. The expression of new pMHC I in cells infected with microbial pathogens or in transformed cells allows the antigen receptors of CD8+ T cells to identify those cells as foreign and to eventually cause their elimination [1], [2], [3]. The effectiveness of immune surveillance however, depends upon the ability of cells to rapidly generate appropriate pMHC I. Timing is particularly important during virus infections to ensure that infected cells are detected before viral progeny are released. The antigen processing pathway for generating pMHC I is therefore required to be comprehensive as well as expeditious.

The MHC I antigen processing pathway does not distinguish self- from non-self proteins: virtually all proteins are used to generate a vast array of pMHC I on the cell surface [1]. Initially, intracellular polypeptides are fragmented by the proteasome in the cytoplasm [4], [5]. The mixture of proteolytic intermediates is actively transported into the endoplasmic reticulum (ER) [6], [7]. In the ER, the antigenic fragments are further trimmed and loaded onto the MHC I molecules which chaperone the peptides to the cell surface [8], [9], [10]. While evidence for the later steps is compelling, the initial entry of polypeptides into the antigen processing pathway is not well understood.

Cells contain thousands of different proteins, in distinct intracellular compartments and at varying levels of abundance. Among all these possible sources, newly synthesized polypeptides are used more efficiently as antigenic precursors [11], [12], in comparison to polypeptides undergoing normal turn-over [13]. Several independent studies have provided evidence linking protein synthesis with pMHC I presentation [14], [15], [16]. The preference of “new” over “old” could allow the antigen processing pathway to rapidly sample a wide array of endogenous proteins soon after their synthesis for presentation as pMHC I. This selection criterion nevertheless poses a conundrum. Most newly synthesized polypeptides are destined for folding and normal biological functions. Yet, a fraction of translated polypeptides enter the mutually exclusive antigen processing pathway for fragmentation into peptides for presentation as pMHC I.

The newly synthesized polypeptides which serve as substrates for antigen processing have been termed DRiPs for defective ribosomal products [17]. DRiPs could be marked for proteasomal degradation because they contain inevitable errors in translational or post-translational processes for producing normal functional proteins. Alternatively, it has been hypothesized that a novel set of “immunoribosomes” may specialize in producing DRiPs as unique substrates for antigen processing [18]. While attractive from the vantage of immune surveillance, the nature of DRiPs remains unknown. Furthermore, it has been difficult to distinguish which cohort of newly synthesized polypeptides serve as precursors for antigen processing versus those with normal biological functions [18], [19].

We reasoned that if DRiPs are an important source of pMHC I used for immune surveillance, analysis of DRiPs might be possible during synthesis of viral proteins. The Epstein-Barr Virus (EBV) is a gammaherpesvirus well known for its ability to establish life-long latency [20]. Despite EBV's quiescence, infected cells express the EBNA1 protein to maintain the viral episome. EBNA1 is therefore a hallmark of EBV latency and EBV associated cancers as well as a potential source of pMHC I [21]. Notably, EBV evades immune surveillance because peptides derived from wild-type EBNA1 are presented poorly by MHC I on the cell surface due to presence of a stretch of glycine-alanine codons (GAr) [22], [23], [24], [25]. The GAr could affect pMHC I presentation by inhibiting proteolytic activity of the proteasome [26], [27]. Alternatively, because it inhibits translation of EBNA1, GAr could also affect generation of DRiPs used for antigen presentation [28], [29], [30]. These hypotheses remain unresolved because the putative DRiPs produced during EBNA1 translation have not yet been identified.

DRiPs as evanescent substrates for antigen processing are difficult to detect using conventional methods. We recently described a novel strategy to detect otherwise invisible antigenic precursors and proteolytic intermediates in the antigen processing pathway [6], [7], [31]. The proteolytic intermediates, containing antigenic peptides with N-and C-terminal flanking residues, are normally inactive in conventional T cell assays. However, by enzymatically releasing the T cell activating peptide from inactive polypeptides, the antigenic precursors can be readily analyzed in cells.

Here we analyzed the translation and processing of the EBNA1 protein associated with EBV latency. We found that ribosomes correctly initiate EBNA1 mRNA translation *in vitro* but rapidly produce truncated antigenic precursors or DRiPs. The mRNA sequence coding for the GAr motif is responsible for the interruption of EBNA1 translation. These DRiPs are rapidly produced in cells but their amount is down-regulated compared to antigenic precursors generated in the absence of GAr. Thus, translation of viral mRNAs as truncated polypeptides is important for determining the antigenicity of virus proteins.

## Results

### GAr inhibits protein synthesis without interfering with translational initiation of EBNA mRNA *in vitro*

**Figure 1:**
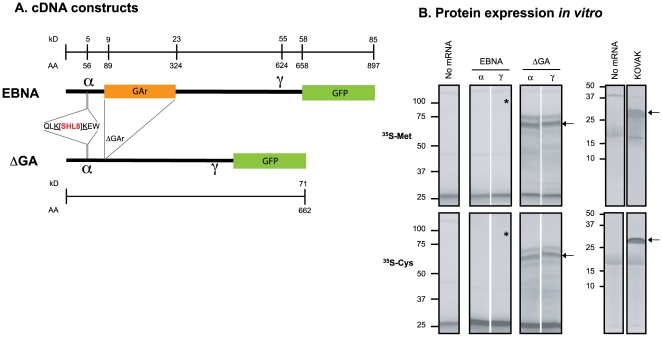
Schematic representation of cDNA constructs encoding EBNA1 and its EBNA1ΔGA derivative lacking the glycine-alanine repeat (GAr). To track the translated products, the OVA-derived epitope (SIINFEHL or SHL8) was inserted in either the N-terminal (positionα) or C-terminal region (positionγ) of EBNA-GFP or its ΔGA derivative. The SHL8 antigenic peptide was flanked at its N- and C-termini by lysines (QLK- and –KEW) to allow detection of potential antigenic intermediates containing the SHL8 peptide by enzymatic methods. The number of amino acids and the predicted molecular weights of putative polypeptides are shown. (**B**) ***In vitro*** **expression EBNA1 and EBNA1ΔGA full-length proteins.** The mRNAs encoding EBNA1 (α or γ) and EBNA1ΔGA (α or γ) were transcribed *in vitro*, purified and translated in a rabbit reticulocyte lysate in the presence of radiolabeled ^35^S-Met or ^35^S-Cys as tracers. The translated products were fractionated on 7.5% SDS-PAGE gels and detected by PhosphoImager. As controls, translation was carried out in parallel without mRNA (no mRNA) or with mRNAs encoding the 27kD KOVAK protein. Arrows indicate the predominant products from translation of ΔGAα, ΔGAγ and KOVAK mRNA (73 and 27 kD respectively). The expected location of the 86 kD product of EBNAα and EBNAγ translation is indicated by an asterisk. Autoradiographs on right show the KOVAK translated products fractionated on higher resolution 16.5% SDS-PAGE. Data shown is representative of 3 different experiments.

We used the Epstein Barr Virus (EBV) encoded EBNA1 (EBNA) protein and its derivative EBNA1ΔGA (ΔGA), without the 705 nucleotides encoding the glycine-alanine repeat (GAr) to define the influence of GAr on the synthesis and processing of EBNA1 antigenic precursors in the MHC I antigen presentation pathway. To detect expression of full-length proteins we used cDNAs encoding the wild-type EBNA1 or the EBNA1ΔGA proteins fused to the green fluorescent protein (GFP) [30] (**Fig. 1A**). To follow the fate of the protein during antigen processing, we inserted nucleotides encoding the modified ovalbumin peptide, referred to as the “KOVAK cassette” in-frame within the EBNA sequences [7]. The KOVAK cassette encodes the ovalbumin derived SHL8 (SIINFEHL) peptide with lysine residues (K) flanking its N- and C-termini (QLK-[SHL8]-KEW) which enable tracking the otherwise undetectable antigenic precursors in the antigen processing pathway. These EBNA1 and EBNA1ΔGA cDNA constructs are referred to as EBNAα or ΔGAα when the KOVAK cassette is inserted in position α, and as EBNAγ or ΔGAγ, when the KOVAK cassette is inserted in position γ.

We first analyzed translation of these mRNAs in an *in vitro* assay. The mRNAs were used as templates for protein synthesis in a rabbit reticulocyte lysate (RRL) with ^35^S-Met or ^35^S-Cys as radiolabeled tracers. After 60 min., the products were fractionated on SDS-polyacrylamide gels and analyzed by autoradiography (**Fig. 1B**). Radiolabeled polypeptides were not detected without addition of mRNA, while KOVAK mRNAs were translated into the expected 27kD product. Likewise, high-molecular weight bands with varying intensities were detected with EBNA1ΔGA mRNAs translated with either ^35^S-Met or ^35^S-Cys tracers. The largest of these bands corresponds to the expected 73kD full-length ΔGA-GFP fusion protein (see **Fig. 1A**). Notably, translation of EBNA1 mRNAs, was similar to the no mRNA control with no detectable polypeptides with either tracer. Thus the presence of GAr was clearly deleterious for translation of EBNA1 mRNA *in vitro* [30]. Note that the negative effect of GAr was independent of the α or γ location of SHL8 within the coding sequence.

**Figure 2:**
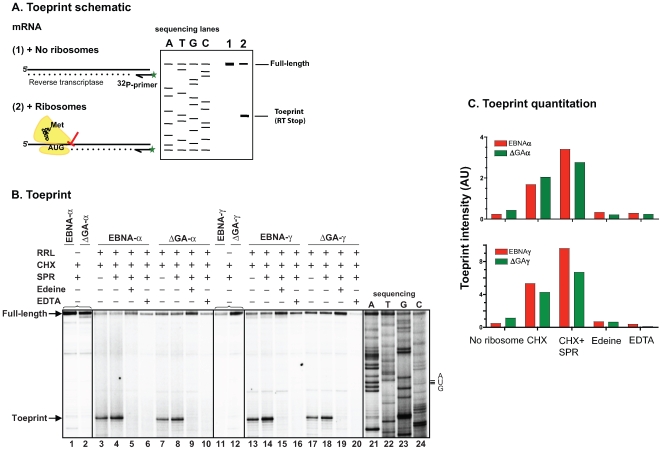
GAr inhibits overall translation of EBNA1 mRNA without affecting ribosomal initiation. (**A**) Schematic of the primer extension inhibition (toeprint) assay illustrating how presence of ribosomes bound to the mRNA initiation codon is detected by analysis of reverse transcriptase (RT) products of varying length. (**B**) The mRNA encoding EBNA1 (α or γ) and EBNA1ΔGA (α or γ) were used as template for the toe-printing assay. Reactions were carried out with either no ribosomes, or in presence of elongation inhibitors, cycloheximide (CHX), CHX plus sparsomycin (SPR), initiation inhibitor, edeine or magnesium chelator, EDTA. Dried gels were analyzed using a PhosphoImager. The full-length and shorter RT (toeprint) products are indicated by arrows. The toeprint corresponds to 17 nucleotides downstream of the AUG codon as judged by the size of fragments on the sequencing gel shown on the right. (**C**) The intensity of the ^32^P radiolabeled toeprint bands observed under the indicated conditions was quantitated and is shown as arbitrary units (AU.) Data are representative of 3 different experiments.

We considered the likelihood that GAr might have inhibited synthesis of EBNA1 polypeptides by interfering with translational initiation [28], [32]. To test this possibility, we carried out primer extension inhibition (toeprint) analysis to determine whether ribosomes efficiently recognized the AUG initiation codon in the EBNA mRNAs (**Fig. 2**). The mRNAs were added to rabbit reticulocyte lysate (RRL) in the presence of translation elongation inhibitors. Cycloheximide (CHX) or both CHX and sparsomycin (SPR) prevent ribosomal movement during protein translation, but do not interfere with ribosomal binding to the initiation codon. The location of the mRNA bound ribosomes was determined using reverse transcriptase (RT) to extend a ^32^P-labeled reverse primer downstream of the start codon. The ^32^P-labeled RT products were fractionated on urea-acrylamide gels to determine their size in comparison to the bands obtained by sequencing the same mRNAs. In the absence of ribosomes, the only predominant band detected corresponded to the full-length RT product (**Fig. 2B**). By contrast, in the presence of ribosomes and elongation inhibitors, CHX alone or CHX+SPR, a strong band of similar intensity was detected with both EBNA1 and EBNA1ΔGA mRNAs. The size of this band, called the “toeprint”, corresponded to exactly 17 nucleotides downstream of the AUG codon; reflecting the inhibition of RT caused by the leading edge (toe) of the ribosome bound to its P site to the initiation codon in the mRNA. As further controls, the toeprints were not detected in the presence of translation initiation inhibitors like edeine or bruceantin, nor when the ribosomes were disrupted by depleting magnesium ions with EDTA (**Fig. 2B**). Importantly, the pattern of the toeprint was indistinguishable between EBNA and ΔGA showing that the GAr region does not influence the ribosome initiation events (**Fig. 2C**). The formation of the initiation complex was also independent of the location of the SHL8 coding sequence within EBNA as shown by identical toeprints with EBNAα, EBNAγ, ΔGAα and ΔGAγ mRNAs. Thus, ribosomes recognized the AUG initiation codon with comparable efficiency in both EBNA and ΔGA mRNAs.

### Ribosomes terminate EBNA translation prematurely

**Figure 3:**
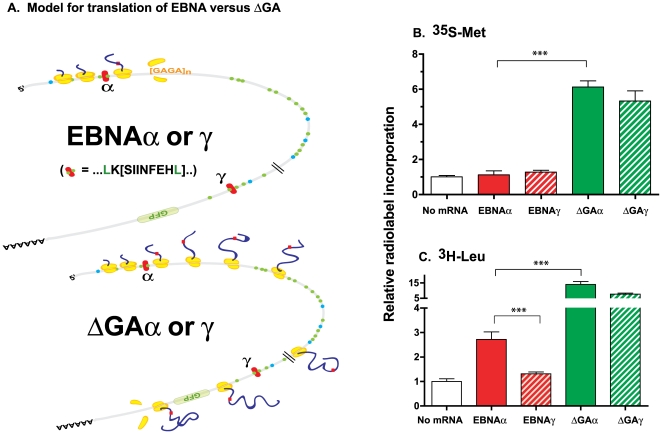
Ribosomes prematurely terminate translation within the 5′ region of EBNA mRNA. (**A**) Schematic model to account for relative differences in the translational efficiency of EBNA or ΔGA mRNAs. The ribosomes initiate translation at the AUG codon (5′ blue dot) of either EBNA or ΔGA mRNAs. However, translation is terminated within or close to the GAr region shown as [GAGA]n in the EBNA mRNA. The methionine and leucine codons are indicated as blue or green dots respectively. The inserted SHL8 nucleotides, at either the N-terminal “α” or the C-terminal “γ” positions are shown as a red rectangle. All constructs included the indicated in-frame green fluorescent protein (GFP) sequence. (**B**) The mRNA coding for EBNAα, ΔGAα, EBNAγ or ΔGAγ were translated *in vitro* in the presence of ^35^S-Met or ^3^H-Leu for 1 h. Translation products were precipitated with trichloroacetic acid and collected by filtration. Radioactivity measured by liquid scintillation is shown as relative counts per minute compared to no mRNA control. Bars are average of 5 different experiments (± standard deviation). Standard *t*-test was used to calculate statistically significant differences shown as asterisks (***: p<0.001).

Next, we addressed the possibility that despite appropriate initiation, the ribosomes could have terminated protein synthesis prior to reaching the authentic termination codon. Such prematurely terminated polypeptides would be difficult to detect as discrete bands on SDS-PAGE gels because of their smaller size as well as possible heterogeneity. Examination of the coding potential of EBNA1 or EBNA1ΔGA mRNA showed a total of 11 Met and 40 Leu residues (**Fig. 3A** and **Supp Fig S1**). Notably, the 5′ sequence preceding the GAr contained only a single Met, encoded by the AUG initiation codon, as well as three Leu residues two of which were encoded by the “KOVAK” cassette inserted in the “α” position (QLK-[SHL8]-KEW, **Fig. 1A**). We used mRNAs for EBNA and its ΔGA derivative containing the KOVAK cassette in the “α” or in the “γ” position downstream of GAr as templates for translation *in vitro* (**Fig. 3A**). To detect putative translation products regardless of their size or heterogeneity, we analyzed the radiolabeled material after precipitating the polypeptides with 25% trichloroacetic acid. As expected from the SDS-PAGE analysis, ^35^S-Met was efficiently incorporated in the TCA precipitated material translated from either ΔGAα or ΔGAγ mRNAs (**Fig. 3B**). However, ^35^S-Met incorporation in products of EBNAα or EBNAγ mRNAs was indistinguishable from the no mRNA control. Likewise, when ^3^H-Leu was used as a tracer, it was incorporated with high efficiency in polypeptides translated from either ΔGAα or ΔGAγ mRNAs (**Fig. 3C**). However, incorporation of ^3^H-Leu in translation products of EBNAα, although lower than in ΔGAα products was significantly above the no mRNA control (p<0.001). In contrast, ^3^H-Leu was not incorporated above the no mRNA background when EBNAγ mRNA was translated despite efficient recognition of its initiation codon by the ribosomes (**Fig. 2**). Thus, the higher incorporation of ^3^H-Leu in EBNAα compared to EBNAγ correlated with the position of the KOVAK cassette relative to the GAr. We conclude that the EBNAα mRNA was translated into polypeptides containing Leu residues although the overall efficiency was clearly lower than with ΔGAα or ΔGAγ. Most importantly, changing the location of the KOVAK cassette, including its two Leu residues, downstream of GAr abrogated ^3^H-Leu incorporation. These results showed that translation of EBNA mRNA did occur but was terminated upstream or within the GAr region.

### Truncated translation products are antigenic precursors

**Figure 4:**
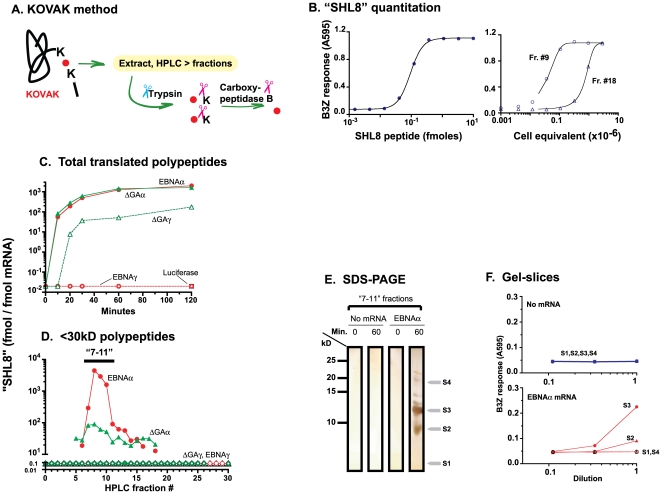
Truncated translation products are antigenic precursors. (**A**) Schematic illustration of the KOVAK method for detection and quantitation of proteolytic intermediates. The SHL8 (SIINFEHL) codons (red circle) are flanked at the N- and C-termini by lysine (K) residues. The putative products of translation are collected in 10% acetic acid before HPLC fractionation. Each fraction is treated with trypsin and carboxypeptidase B (CPB) to release the optimally active SHL8 peptide. (**B**) The amount of embedded SHL8 in the proteolytic intermediates (“SHL8” activity) is shown in two representative HPLC fractions (#9 and #18). The B3Z stimulating activity in dilutions of fraction #9 and #18 was compared with a standard curve generated with synthetic SHL8 peptide. (**C**) The indicated mRNA coding for EBNAα, EBNAγ, ΔGAα, ΔGAγ and luciferase as a negative control, were translated *in vitro* for varying time periods. Polypeptides in aliquots of the translation reactions were extracted assayed without HPLC fractionation as in (A). (**D**) Products translated in 60 min. *in vitro* from the indicated mRNAs were passed through a 30kD molecular weight filter. The filtrate (<30kD) was fractionated by HPLC and the amount of “SHL8” activity in each fraction was determined. Data shown represent 3 different experiments. (**E**) Polypeptides translated from EBNAα mRNA and eluting in “7–11” HPLC fractions in (C) were fractionated on high resolution tricine 16.5% SDS-PAGE gels. “No mRNA” samples were analyzed in parallel as negative controls. The silver-stained gel shown is representative of 3 different experiments. (**F**) Indicated slices (S1–S4) were excised from the gel, treated with trypsin and CPB and dilutions were tested for “SHL8” activity with SHL8/K^b^-specific B3Z lacZ inducible T cell hybridoma and K^b^-L cells as APC. The conversion of the lacZ substrate chlorophenol β-D-pyrannoside was measured as absorbance at 595 nm with 655 nm as reference wave length. Data are representative of 3 different experiments.

To directly characterize the translated polypeptides, we took advantage of the KOVAK cassette which contains the antigenic SHL8 peptide flanked by two lysine residues. We have shown earlier that polypeptides containing the KOVAK cassettes can be detected with high sensitivity [7]. This strategy is based on the fact that even sub-femtomolar amounts of the SHL8 peptide can be readily detected by B3Z T cells in presence of K^b^-APC. However, longer polypeptides which contain SHL8 in an internal position remain undetectable because they are inactive in this assay. This profound limitation in sensitivity can be overcome by enzymatically releasing the exact SHL8 peptide from its embedded location within the polypeptide sequence. The optimally active SHL8 peptide can be released from the precursor polypeptide by trypsin and carboxypeptidase B (CPB) due to the two lysine residues flanking the SHL8 peptide (**Fig. 4A**). The polypeptides can thus be detected by their “SHL8 activity” and quantitated by comparison with a standard curve generated with synthetic SHL8 peptide (**Fig. 4B**).

With mRNAs encoding ΔGAα or EBNAα as templates in an *in vitro* translation assay, SHL8 polypeptides were detected within 10 minutes after initiation of translation and reached a peak in 60 minutes (**Fig. 4C**). No SHL8 was detected in translation products of luciferase mRNA, used as a negative control. Given that ΔGAα is translated as a large, full-length protein (**Fig. 1B**), and EBNAα translation is prematurely terminated (**Fig. 3**), similar “SHL8” activity recovered from both reactions suggests that translation of the 5′ region of these mRNAs occurred with similar efficiency. Likewise, when the SHL8 peptide was located in the 3′ “γ” location downstream of GAr (**Fig. 1**), the SHL8 polypeptides were produced from ΔGAγ mRNA but were not detected when EBNAγ mRNA was translated. These results independently establish that distinct regions of mRNA were translated with different efficiencies in the presence of GAr. The translation of the SHL8 sequence when present in the 5′ “α” location occurred efficiently with or without GAr. However, when SHL8 was located in the 3′ “γ” location, the upstream GAr strongly inhibited the translation of SHL8 containing polypeptides demonstrating that translation was effectively terminated upstream of the SHL8 codons.

We characterized the translation products further by HPLC fractionation. The *in vitro* translated polypeptides were acid extracted, passed through a 30kD filter, and the <30kD filtrate was fractionated by reverse phase HPLC (**Fig. 4D**). Each HPLC fraction was treated with trypsin and CPB and analyzed for presence of SHL8 activity. As expected, no SHL8 activity was detected in HPLC fractionated material translated from EBNAγ mRNA encoding SHL8 in the 3′ region. However, when EBNAα mRNA was translated, SHL8 polypeptides eluted in a broad peak with >90% of total activity in fractions 7–11. In contrast, only 3% of SHL8 polypeptides eluted in the same 7–11 fractions in translated products of ΔGAα mRNA. We infer that the majority of ΔGAα antigenic products (**Fig. 4C**) were larger than 30kD. This inference is also in agreement with the absence of ΔGAγ mRNA translation products in 30kD extracts, consistent with the 5′ position of SHL8.

To independently estimate their length, we pooled the “7–11” fractions and separated the polypeptides on tricine SDS-PAGE gels (**Fig. 4E**). Six bands, ranging in size from 8–20 kD were detected in the material after 60 minutes of EBNAα mRNA translation. The ∼12 kD band was the most prominent, followed by the band at ∼8 kD. Gel slices (S1–S4) containing these two as well other bands were excised from the gel and tested for SHL8 activity after treating the eluted material with trypsin and CPB (**Fig. 4F**). Slice S3, containing the 12 kD band had the most SHL8 activity compared to the other slices. On the other hand, SHL8 activity was not detected in slices from the no mRNA control gel. Assuming no post-translational modifications, 12 kD corresponds to approximately 120 residues in EBNAα (**Fig. 1A**). Interestingly such polypeptides also include 19 glycine-alanine residues consistent with termination of translation shortly after entry of ribosomes into the GAr coding sequence. We conclude that translation of EBNAα is terminated prematurely to produce truncated polypeptides which fit the definition of defective ribosomal products or DRiPs as originally proposed by Yewdell and colleagues [17].

### Truncated polypeptides are generated in living cells

To determine if similar translation products were generated in living cells, the cDNAs were cloned in a tetracycline regulated vector and stably transfected into the Tet-ON human embryonic kidney cells (HEK293). Measurement of GFP fluorescence by microscopy or flow cytometry showed that protein expression was induced upon the addition of the tetracycline analog, doxycycline (**Supp Fig S2**). In contrast to cells transfected with vector alone, EBNAα, EBNAγ, ΔGAα and ΔGAγ transfected cells expressed GFP fluorescence as early as 3 h after doxycycline addition and reached a plateau after 24 h. Moreover, as reported earlier [30] and similarly to our *in vitro* observations, GFP expression in cells expressing full-length EBNA1 was substantially lower than in cells expressing EBNA1ΔGA lacking GAr. The difference in protein expression was not due to differential transcription, because the cell lines contained similar amounts of mRNA measured by quantitative RT-PCR (data not shown). Thus protein expression was inducible *in vivo* and regulated by GAr in EBNA1 precursors.

**Figure 5:**
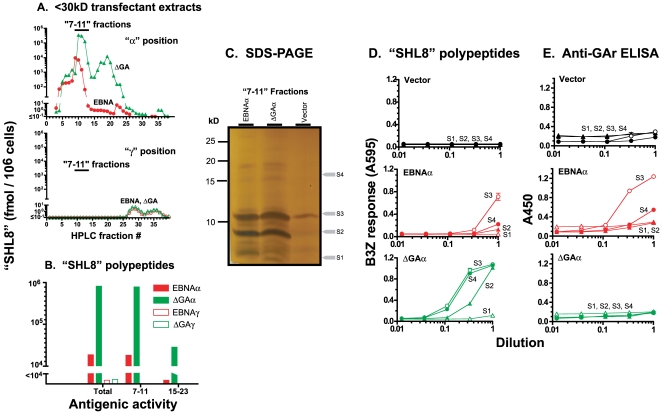
Generation of DRiPs in living cells. (**A**) HEK293 cells were induced to express EBNA or ΔGA with SHL8 in position “α” (upper panel) or “γ” (lower panel) with doxycycline for 24 h. The cell extracts were passed through 30kD molecular weight filters and the <30kD filtrates were fractionated by HPLC. Each HPLC fraction was treated with trypsin and CPB and the amount peptides containing “SHL8” was determined. (**B**) The total amount of “SHL8” activity recovered in the polypeptides eluting in all HPLC fractions versus fractions “7–11” and “15–23”. Data represent 3 different experiments. (**C**) HEK293 cells expressing vector alone, EBNAα or ΔGAα were cultured for 24h with doxycycline. The cell extracts were passed through 30 kD molecular weight filters and fractionated by HPLC. The polypeptides in fractions “7–11” were pooled and separated on tricine 16.5% SDS-PAGE. The silver-stained gels shown is representative of three different experiments. (**D**) The indicated slices (S1–S4) were cut from the gel and digested for 8 h by trypsin. Each digested slice was split into two aliquots. Aliquots from the indicated vector, EBNAα or ΔGAα samples were further digested with CPB for 4 h. The “SHL8” activity was measured in the indicated dilutions using B3Z T cells and K^b^-L cells as APC. The B3Z response is shown as products of the lacZ substrate CPRG as in Fig. 4. (**E**) The presence of the GAr motif was detected by an ELISA assay using a mouse anti-GAr antibody and anti-mouse conjugated to horse-radish peroxidase. Data shows the absorbance (A450) of the peroxidase substrate converted in 30 min.

To analyze processing intermediates, protein expression was induced by treating the stable transfectants with doxycycline. The polypeptides were acid-extracted and passed through a 30 kD molecular weight filter and the filtrate was fractionated by HPLC. Each fraction was then assayed for SHL8 polypeptides after treatment with trypsin and CPB as above (**Fig. 5A**). In EBNAα expressing cells, ∼96% of the SHL8 polypeptides, similar to those produced *in vitro*, eluted in the “7–11” fractions. Surprisingly, although the <30 kD SHL8 polypeptides were generated poorly *in vitro* after translation of ΔGAα mRNA, the extracts of cells expressing ΔGAα contained 10–40 fold higher amount of SHL8 activity in the <30 kD material compared to cells expressing EBNAα (**Fig. 5A,B**). Approximately 95% of SHL8 polypeptides eluted in the same “7–11” fractions and remaining ∼5% were found in fractions 15–23. Notably, no SHL8 activity was detected in <3 kD filtrates or when the HPLC fractions were assayed in PBS, trypsin or CPB alone confirming that the SHL8 containing polypeptides were between 3–30 kD in size and contained both N- and C-terminal residues flanking the antigenic peptide (**Supp Fig S3**). In contrast, similar <30 kD extracts of EBNAγ or ΔGAγ expressing cells did not contain SHL8 polypeptides eluting in the same HPLC fractions and only a small amount of activity was detected in fractions 27–37 (**Fig. 5A, lower panel**). Because HPLC elution profiles reflect the biochemical properties of the polypeptides, we infer that the <30kD SHL8 polypeptides generated from the “α” versus “γ” locations in the mRNA were structurally distinct [31].

To confirm the structural features of DRiPs produced in living cells, we analyzed the pooled “7–11” HPLC fractions by SDS-PAGE as with the *in vitro* translation products above (**Fig. 5C**). In silver stained gels, at least ten bands were detected ranging in size from less than 10 kD to 20 kD in cells transfected with the vector alone and in higher amounts in cells stably transfected with the ΔGAα or EBNAα cDNA constructs. As *in vitro* translation assays, the most prominent bands had molecular weights of approximately 12 and 8 kD but the intensity of the bands was comparable for polypeptides extracted from EBNAα and ΔGAα transfected cells. This result is consistent with the expectation that in contrast to *in vitro* translation of single mRNA molecule, transfected cells would also contain various other polypeptides which elute in the same “7–11” HPLC fractions.

The material available in the gels was insufficient for mass-spectrometric analysis. We therefore characterized the polypeptides contained in the bands by eluting the material from four gel slices representing predominant as well as less prominent bands (S1–S4, **Fig. 5C**). The eluted polypeptides were tested for presence of SHL8 activity after trypsin and CPB treatment as well as GAr epitopes with ELISA (**Fig. 5D,E**). In gel slices of vector alone samples, neither SHL8 nor GAr activity was detected. In contrast, SHL8 activity was detected in the ∼12 kD S3 slice but not in the other three slices from the EBNAα sample (**Fig. 5D**). Interestingly, the ∼12kD (S3) and the ∼18kD (S4) slices from the EBNAα sample also contained GAr epitopes as predicted by the sequence of polypeptides with these sizes (**Fig. 1A** **,** **5E**). Notably, in the ΔGAα sample, three slices (S2, S3 and S4) representing ∼8, ∼12 and ∼18kD polypeptides contained substantial SHL8 activity. However, none of these slices contained polypeptides with GAr epitopes as expected for the ΔGAα construct with deleted GAr. Taken together these results established that cells expressing EBNAα or ΔGAα constructs translate a heterogeneous mixture of DRiPs as antigenic precursors. Further, the structure and amount of DRiPs was determined by the presence of GAr coding sequence.

### EBNA precursors are rapidly processed to the final peptide

**Figure 6:**
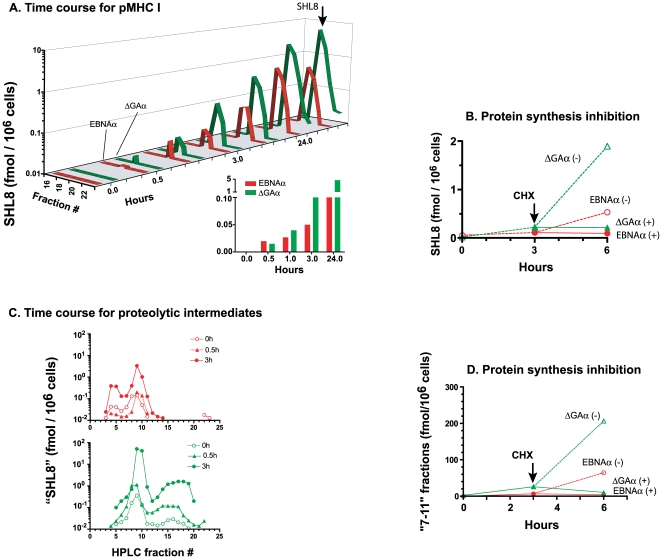
Newly synthesized precursors are processed rapidly to the final SHL8 peptide. (**A**) HEK293 cells expressing K^b^ MHC together with EBNAα (red) or ΔGAα (green) were treated with doxycycline. At the indicated time points, peptides were extracted from the cells and fractionated by reverse phase HPLC. The fractions were assayed without any further treatment using B3Z T cells and K^b^-L cells as APC and quantitated using synthetic SHL8 as a standard (upper panel). The total SHL8 recovered at different time points is indicated in lower panel. Data are representative of 3 different experiments. (**B**) Protein expression was induced in the same cells with doxycycline for 3 h. The cells were treated (+) with the protein synthesis inhibitor cycloheximide or used as such (−). The total amount of SHL8 eluted in fractions 17 to 22 was measured at 0 h, 3 h and 6 h. Arrow indicates addition of cycloheximide. Data shown are typical of three different experiments. (**C**) HEK293 cells expressing EBNAα (red) or ΔGAα (green) were treated with doxycycline for the indicated times. The polypeptides were extracted and “SHL8” activity was quantitated as described in Fig. 4. Total “SHL8” activity in fractions “7–11” are presented in lower panel. Data is from three different experiments. (**D**) Same cells were stimulated 3 h with doxycycline following same treatments than in B. The “SHL8 activity” in fractions “7–11” was measured at 0 h, 3 h and 6 h, in the presence or not of cycloheximide. Arrow indicates addition of drug. Data shown are typical of three different experiments.

Finally, we determined the temporal relationship between the generation of DRiPs and the appearance of the final processed pMHC I. Peptides were extracted from cells expressing either full-length EBNAα or its ΔGAα derivative after varying times and fractionated by HPLC. To assay only the final processed SHL8 peptide, the fractions were assayed as such without any enzymatic treatment (**Fig. 6A**). A single peak of antigenic activity, co-eluting with the synthetic SHL8 peptide in HPLC fractions #18–20 was detected as early as 30 minutes after inducing gene expression with doxycycline and increased steadily over the next 24 h. We estimated that EBNAα expressing cells produced about 0.02 and 1 fmol per million cells at 30 min and 24 h respectively. On a per cell basis, this amount corresponds to 12 copies of SHL8 peptide which increased to about 600 copies at 24 h. Notably, in the same time period, the ΔGAα precursor yielded 5–10 fold higher amount of processed SHL8 peptide relative to its full-length EBNAα counterpart. Note that no SHL8 was detected in the absence K^b^ indicating that SHL8 quantification corresponds to the formation of the pMHC I (data not shown).

The EBNA1 as well as its EBNA1ΔGA derivatives are stable long-lived proteins with half-lives of over 20 hours [24]. The rapid generation of pMHC I suggested that the processed peptides were most likely derived from newly synthesized precursors rather than turn-over of stable EBNA1 or EBNA1ΔGA proteins. To test this hypothesis, we treated cells with cycloheximide, a protein synthesis inhibitor, for three hours after inducing protein synthesis with doxycycline. The amount of processed SHL8 peptide generated in the cells was then estimated in cell extracts after HPLC fractionation (**Fig. 6B**). In contrast to the accumulation of the processed SHL8 peptide in untreated cells (−), cycloheximide treatment (+) completely blocked the generation of SHL8 in EBNAα or ΔGAα expressing cells. In an independent approach that did not require inhibitors, we examined the generation of SHL8 peptide in cells after removing doxycycline which is required for protein expression (**Supp Fig S4**). The EBNAα or ΔGAα expression was induced with doxycycline for 24 h and the cells were briefly treated with mild-acid. As judged by their ability to stimulate B3Z T cells, the acid-wash removed the pre-existing SHL8-K^b^ complexes (**Supp Fig S4A**). To distinguish whether the processed peptides were derived from newly synthesized or pre-existing antigenic precursors, we measured the recovery of SHL8-K^b^ complexes in the absence or presence of doxycycline (**Supp Fig S4B, C**). Flow cytometry measurements showed that about 86% of EBNAα and ∼99% of ΔGAα transfected cells were GFP+ indicating that these antigenic proteins were present in the cells (**Supp Fig S4B**). Nevertheless, in the absence of doxycycline, the EBNAα and ΔGAα cells generated only about 30% processed SHL8 compared to cells cultured in the presence of doxycycline (**Supp Fig S4C**). Thus, most of the processed SHL8 peptide was clearly derived from newly synthesized precursors.

To establish that precursor “SHL8” polypeptides were generated in the same time frame, we treated HEK293 cells expressing EBNAα or ΔGAα with doxycycline for 3 h and assayed the “SHL8 activity” of in cell extracts as above. The fractions “7–11” SHL8 polypeptides were generated within 0.5 h after doxycycline addition in ΔGAα and EBNAα expressing cells (**Fig. 6C**). Nevertheless, DRiPs were generated at a slower rate and in substantially lower amounts from EBNAα containing the GAr sequence. In both EBNAα and ΔGAα expressing cells, the SHL8 polypeptides detected in fractions “7–11” rapidly decreased when protein synthesis was arrested (**Fig. 6D**). Thus, new protein synthesis is required for rapid production of the “7–11” polypeptides.

## Discussion

We have shown here that cells synthesize truncated polypeptides during translation of EBNA mRNA as potential antigenic precursors. The appearance of these polypeptides reveals that translation of viral mRNA yields not only the full-length protein, but also the elusive defective ribosomal products referred to as defective ribosomal products or DRiPs. Furthermore, because DRiPs were produced rapidly and their expression was markedly inhibited by the glycine-alanine repeat in EBNA, suggests that this could be a novel mRNA translation mechanism for evading immunity.

DRiPs were first proposed to serve as a primary source of antigenic peptides presented by MHC I more than a decade ago by Yewdell and colleagues [17]. The DRiPs hypothesis has since been supported by several independent studies [11], [12], [15], [16]. The key advantage of using DRiPs rather than normal protein turn-over as a source of antigenic peptides is that infected cells can use DRiPs to present pMHC I rapidly regardless of full-length protein turn-over. The emergence of virus derived pMHC I on the cell surface would permit cytotoxic T cells to detect infected cells before viral progeny are released. Although attractive from an immunological perspective, the molecular characteristics of DRiPs are not known and their relationship to normal protein synthesis and turn-over is poorly understood [18], [19].

Polypeptides can be defective due to errors that might occur during or after translation. For example, ribosomes could initiate or terminate translation at inappropriate locations in the mRNA [33], or mis-translate codons using wrong amino acids [34]. In addition, full-length normal polypeptides could also be considered defective due to problems in folding or incorporation into multi-subunit complexes [35]. Because defective polypeptides are rapidly degraded by the proteasome they could serve as significant source for antigenic peptides [36]. Nevertheless, such defective polypeptides have been difficult to analyze by conventional methods likely due to their transience and miniscule amounts.

We used several different approaches to characterize the antigenic precursors produced by translation of EBNA and ΔGAr mRNAs (Fig. 1). As noted earlier, full-length EBNA compared to ΔGA mRNA (more clear to say mRNA is translated, not protein) was translated at a remarkably low efficiency [24], [28]. Yet, the ribosomes recognized the appropriate AUG initiation codon in both EBNA and ΔGA mRNAs with similar efficiency. Thus, the translation initiation step, a frequent target for regulating protein synthesis [37], could not account for differences in translation of these mRNAs. Instead, we found that the ribosomes prematurely terminated translation of EBNA mRNA *in vitro*. Incorporation of radiolabeled amino acids in the *in vitro* synthesized material revealed that the majority of ribosomes translated only the 5′ region of EBNA mRNA. Furthermore, analysis of EBNA translation with the KOVAK method which allows detection of rare antigenic precursors, revealed that the most predominant EBNA polypeptides were heterogeneous and contained ∼120 residues.

Truncated polypeptides were also produced rapidly in living cells from both EBNAα and ΔGAα albeit in vastly different amounts. The amount of DRiPs produced in cells expressing EBNAα was less than 10% compared to DRiPs produced in cells expressing ΔGAα (Fig. 5). The reason why cells produce such different amounts of DRiPs in the presence or absence of ΔGAr in living cells is not clear. Regardless, the DRiPs eluting in “7–11” HPLC fractions contained approximately 95% of antigenic activity and the “15–23” fractions contained the remainder. Although the identity of the small amount of polypeptides eluting in “15–23” fractions is not yet known, SDS-PAGE analysis showed that the predominant polypeptides in the “7–11” fractions produced from either EBNAα or ΔGAα were similar; approximately 120 residues in length. Furthermore, consistent with the presence of the unique GAr region, only the DRiPs in EBNAα expressing cells contained the GAr epitopes. Notably, the DRiPs in “7–11” HPLC fractions were specific to EBNAα and ΔGAα which contained the SHL8 peptide in the “α” location. These polypeptides were not found in cells expressing EBNAγ or the ΔGAγ constructs. Because HPLC elution profiles reflect the biochemical composition of the polypeptides and are detected by their antigenicity, it is presently unclear whether DRiPs of similar lengths are produced from other regions of the mRNAs as well.

The production of truncated DRiPs and regulation of their amount by GAr sequence shows that antigen presentation can be regulated at the level of mRNA translation. The length of truncated polypeptides places the translation termination site just before or within the GAr region in the EBNA sequence (Fig. 1). Interestingly, the GAr coding sequence shows a marked bias in codon usage [38]. Although any one of four codons can be used to specify the glycine (GGU, GGA, GGC, GGG) or the alanine (GCU, GCA, GCC, GCG) amino acids, the GAr is encoded almost exclusively by codons containing purines for the glycine (GGG, GGA) and alanine (GCA) residues. In an elegant study, Tellam and colleagues recently showed that silent substitution of purines with pyrimidines in the GAr coding sequence enhanced EBNA protein synthesis as well as antigen presentation[29]. Thus, it is plausible that secondary structure of GAr mRNA impedes ribosomal progress and explains how translation of EBNA terminates in the vicinity of GAr sequence.

Regulating production of DRiPs at the level of mRNA translation may serve as an immune evasion strategy for latent viruses. Gammaherpesviruses, such as Epstein-Barr Virus (EBV) or Kaposi's Sarcoma-Associated Herpesvirus (KSHV) infect humans as persistent life-long infections [39]. Yet, expression of EBNA1 or LANA1 latency proteins is essential for maintenance of the EBV or KSHV viral episomes in host cells. Like EBNA1, LANA1 also contains a “QED” repeat region which is implicated in blocking presentation of pMHC I [40]. It is tempting to speculate that episome maintenance proteins, found in herpesviruses of various species [41], [42], might have evolved to inhibit pMHC I presentation by interfering with production of DRiPs. Knowing that DRiPs can be defined as truncated polypeptides provides insights into structure of DRiPs and opens the door to understanding regulation of the pMHC I repertoire in latent viruses.

## Materials and Methods

### Plasmid constructs

The pEGFP-N1 vectors containing the full-length EBNA1 or EBNA1 without GAr (ΔGA) were a kind gift of R. Khanna and J. Tellam (Queensland Institute of Medical Research, Australia)[24]. The *Not* I site of pEGFP-N1 was deleted by mutagenesis (5′ -GTG GCC GC) following directions provided with the QuikChange Site-Directed Mutagenesis Kit (Stratagene). The KOVAK cassette (QLK[SIINFEHL]KEW) was produced by PCR on overlapping primers and cloned into *Bbs* I within EBNA1 (position α) or *Not* I within the FLR epitope (position γ) using Rapid DNA Ligation Kit (Roche). The primer sequences for PCR were as follows: forward (*Bbs* I), (5′ -CAG CAG AAG ACC AGG AGC GCA GCT GAA GAG CAT CAT CAA CTT CGA GCA CC), reverse (*Bbs* I), (5′ –GGT CAG CTC CTG GTC TTC CCC ACT CCT TCA GGT GCT CGA AGT TGA TGA TGA TG), forward (*Not* I), (5′ -AAG GAA AAA AGC GGC CGC CAG CTG AAG AGC ATC ATC AAC TTC GAG CAC C), forward (*Not* I), (5′ -TTT TCC TTT TGC GGC CGC CCC ACT CCT TCA GGT GCT CGA AGT TGA TGA TG). DNA fragments expressing EBNA1-GFP (α or γ) or EBNA1ΔGA-GFP (α or γ) were obtained by PCR from pEGFP-N1 vectors using a *Hin*d III and *Hpa* I restriction sites. They were cloned into pcDNA5/FRT/TO and pcDNA I through the *Hin*d III and *Eco*R V sites.

### Cell lines

The Flp-ln T-Rex -293 cell line (Invitrogen) referred to as “HEK293” stably expresses the Tet repressor from pcDNA6/TR and contains a single integrated Flp Recombination target (FRT) site from pFRT*/Lac*Zeo. pcDNA5/FRT/TO expressing EBNA1 or EBNA1ΔGA (α or γ) were cotransfected with pOG44 Flp recombinase expression plasmid into Flp-ln T-Rex host cell according to the manufacturer instructions. The gene of interest is integrated in a Flp recombinase-dependent manner into the genome and its expression induced by the doxycycline (Sigma Chemical, 500 ng/mL) to the culture medium for indicated time periods. HEK293 clones were cultured in DMEM medium supplemented with 10% Tet System approved fetal bovine serum (FBS) (Clontech), 25 mM Hepes-Buffer solution (Gibco-BRL), 100 IU/mL penicillin and 100 µg/mL streptomycin (Gibco-BRL), 2 mM L-glutamine (Gibco-BRL), 2 mM sodium pyruvate solution (Gibco-BRL), 2 mM non-essential amino acid solution (Gibco-BRL), 0.5 µM 2-β mercaptoethanol (Sigma Chemical) and blastacidin 10 µg/mL (Invitrogen). After homologous recombination, clones were selected and maintained in the presence of hygromycin B 150 µg/mL (Invitrogen). Clones transfected by pcDNAI/neo expressing H-2K^b^ were selected and maintained in medium containing G418 Sulfate 750 µg/mL (Invitrogen). The culture conditions for K^b^-L fibroblasts and SHL8/K^b^-specific B3Z T hybridoma cells have been described [7]. Analysis of RP-HPLC fractions and peptide titration were performed in RPMI supplemented with 10% Nu-serum (BD Biosciences). Cells were treated with epoxomycin 1 µg/mL (Calbiochem) to inhibit proteasomes function or 50 µM cycloheximide (Sigma Chemical) to inhibit translation activity. Flow cytometry data of GFP+ cells were acquired on an XL Analyzer (Coulter) and analyzed with FlowJo software (Tree Star). For microscopy analysis, HEK293 were cultured 48 h on cover slides pretreated with Poly-L-Lysine Solution 4 h at 37°C (Sigma Chemical) and treated for indicated periods with doxycycline.

### HPLC fractionation

Peptides were extracted from approximately 15×10^6^ cells by boiling in 10% acetic acid solution. After centrifugation for 15 min. at 16,000 g, supernatants were filtered through Microcon filters with a cutoff of 30 kD (Millipore). The filtrate was injected onto a 2.1×250 mm C18 reverse-phase column (Vydac) and separated for 24 min. by a linear gradient of acetonitrile (23–35%) with 0.1% (v/v) trifluoroacetic acid as the ion-pairing agent. Five-drop fractions were collected in flat-bottom 96-well plates and dried in a vacuum centrifuge. Eluted peptides were dissolved in PBS or 20 µg/mL trypsin (Worthington) and Carboxypeptidase B (CPB) 0.1 IU/mL (Worthington), titrated and incubated 6 h at 37°C. SHL8 activity was assayed by adding K^b^-L (4×10^4^ per well) and B3Z T cells (8×10^4^ cells per well) for 18 h in complete RPMI medium with 10% Nu-serum (Thermo Fisher Scientific). B3Z T cell response was assayed as previously described [43].

### *In vitro* transcription and translation

Hpa I linearized pcDNA I plasmids were transcribed for 120 min. at 37°C as previously described [33]. Amount of purified mRNA was measured by absorption at 260 nm. Size and integrity of the mRNA was confirmed by electrophoresis in formaldehyde/MOPS denaturing 1% agarose gels (Ambion). *In vitro* translation was performed using the Flexi Rabbit Reticulocyte Lysate (RLL) System (Promega) as previously described [33] with or without ^35^S-Met (0.4 mCi/mL), ^35^S-Cys (0.3 mCi/mL) or ^3^H-Leu (0.5 mCi/mL). The mRNA coding for luciferase was provided by the RLL system (Promega). Translation reactions were fractionated on 7.5% or 16.5% SDS-PAGE (Biorad) as indicated. Dried gels were exposed on a PhosphoImager screen and analyzed using a Storm PhosphoImager (Molecular Dynamics).

### Trichloroacetic acid (TCA) precipitation

2 µL of the 50 µL *in vitro* translation reaction mixture was added to 98 µL 1 M NaOH/2%H_2_O_2_ and incubated for 10 min. at 37°C. To this mixture, 900 µL of ice-cold 25% TCA/2% casamino acids were added to precipitate the translation products on ice for 30 min. The precipitate was collected by passing through Whatman GF/C glass fiber filters wetted with cold 5% TCA. Precipitated translation products were collected by vacuum filtration, washed 3 times with 3 mL of ice-cold 5% TCA and dried at room temperature. To determine ^35^S-Met or ^3^H-Leu incorporation, radioactivity on the filters was counted in a liquid scintillation counter (Beckmann LS600 IC). For each reaction, the relative incorporation of radiolabel was determined as average (cpm of ppt)/average (cpm of “no mRNA control reaction”).

### Protein bands analysis

HPLC fractions were combined with Tricine sample buffer (5% DTT 1M, 6% glycerol, 2% SDS (w/v), 2.5% G250 1 mg/mL, 2.5% solution Tris·Cl (0.5M)/SDS (0.4% w/v), pH 6.8) and resolved on Tris-Tricine 16.5% SDS-PAGE (Biorad) with Tris-Tricine buffer systems. Resolving gels were either silver stained (PlusOne Silver Staining Kit, GE Healthcare) or stained 2 h with GelCode Blue Stain Reagent (Thermo Scientific) to analyze protein bands. These bands were cut out and washed 20 min. in 500 mL of 100 mM NH_4_HCO_3_ and then 15 min. at 50°C in 100 mL of 100 mM NH_4_HCO_3_ and 10 mL of 45 mM DTT. Ten mL of 100 mM iodoacetamide was added before incubating the solution 15 min. in the dark at room temperature. After discarding the solvent, gel slices were washed in 500 mL of a 50∶50 mix of acetonitrile and 100 mM NH_4_HCO_3_ with shaking for 20 min. at room temperature. After discarding the solvent, 50 mL of acetonitrile was added to shrink the gel pieces. Solvent was removed 15 min. later and gel fragments were dried in speed vacuum. Gel pieces were reswelled 15 min. with 20 mL of 25 mM NH_4_HCO_3_ containing Promega modified trypsin at 20 µg/mL. Thirty mL of additional buffer was added to cover the gel pieces which were incubated 8 h at 37°C. SHL8 activity was measured by addition of CPB as described above. For anti-GAr ELISA, trypsin-digested gel slices solutions were titrated on MaxiSorp flat bottom plates (Nunc) in 0.1 M carbonate buffer [0.032 M Na_2_CO_3_, 0.068 M NaHCO_3_ pH 9.6] and incubated overnight at 4°C. Coated peptides were incubated with PBS 5% milk 2 h at 37°C, washed with PBS 0.1% tween and probed 2 h at 37°C first with anti-GAr Mab at 1 ug/mL (BioVendor Laboratory Medicine, Czech Republic, clone E1-2.5) and a HRP conjugated anti-mouse antibody (Amersham). The signal was revealed by addition of 1-Step Ultra TMB-ELISA substrate (Pierce) 30 min. at room temperature followed by 2 M H_2_SO_4_ (v/v). Absorbance of each well is measured at 450 nm (reference 655 nm).

### RT-qPCR

Total RNA of 10×10^6^ cells was isolated by using TRIZOL Reagent (Invitrogen), treated with RQ1 RNAse-free DNase I (Promega), purified using silica membrane spin columns (Phase Lock Gel, Prime GmbH) and isopropanol precipitated. Four µg of total RNA was reverse transcribed with oligo(dT)15 primers (Promega) using SuperScript III Reverse Transcriptase (Invitrogen). One fmol of purified mRNA transcribed *in vitro* from pcDNA I/EBNAα and pcDNA I/ΔGAα was also reverse transcribed. Their cDNA were used as a quantitative reference during RT-qPCR. SYBR Green PCR was performed with 50 ng and 5 ng of cDNA templates using commercial kit (Applied Biosystems) and GeneAmp 7300 Sequence Detection System (Applied Biosystems). Each sample was analyzed in triplicates, and the amounts of templates were estimated by linear regression against the known standard and normalized to internal control. The primer sequences for the PCR were as follows: EBNA1 Forward, (5′-CTG GAA ATG GCC TAG GAG AG), EBNA1 Reverse, (5′ -CCC TCT TCT TTG AGG TCC AC), human β-Actin Forward, (5′ -GGC ACC ACA CCT TCT ACA ATG), human β-Actin Reverse, (5′-GTG GTG GTG AAG CTG TAG CC).

### Primer extension ‘toeprinting’ assay of initiation complexes

Toeprinting assay was performed as previously described [33]. The 5′-end of the DNA oligonucleotide (5′ -CAT CTG GAC CAG AAG GCT C) used as the reverse primer is complementary to the mRNA at position +64 from the AUG start codon in the pcDNA I vector. Ribosome binding Reaction mixtures were mixed with test compounds 2 µM edeine, 500 µg/mL cyclohexmide, 200 µM sparsomycin, 10 µM EDTA (Sigma)) and template mRNA (0.5 µg/reaction). The reverse transcriptase reaction was mixed with 3.1 pmol ^32^P-primer and 10 IU Avian Myeloblastosis Virus Reverse Transcriptase (RT, Promega). Reactions were layered onto 8% polyacrylamide sequencing gel. Dried gels were exposed on a PhosphoImager screen and analyzed using a Storm PhosphoImager (Molecular Dynamics).

### Statistical analysis

Statistical analysisPrism software (GraphPad) was used for statistical analyses. *P* values were calculated with the unpaired *t*-test.

## Supporting Information

Figure S1 Amino acid sequences encoded by EBNAα-GFP and EBNAγ-GFP cDNA constructs. The GAr sequence is in bold italics and the methionine (M, blue) and leucine (L, green) residues are indicated. Residues of the “KOVAK cassette” inserted in the α or γ positions are highlighted in a box.
(0.42 MB EPS)

Figure S2 HEK293 cells were stably transfected with either vector alone or the indicated EBNA derivatives shown in Fig 1A. Protein expression was induced by addition of doxycycline for the indicated time periods. Fluorescence of GFP was detected by microscopy (A) or as mean fluorescence intensity by flow cytometry (B). Data show mean ± SD of triplicates and are representative of 3 independent experiments.
(4.41 MB EPS)

Figure S3 “SHL8” activity in HPLC fractionated <30kD or <3kD extracts from cells expressing either EBNAα or ΔGAα constructs used in Fig 5. Each HPLC fraction was assayed as such or after treatment with trypsin, carboxypeptidase B (CPB) or both trypsin and CPB. The “SHL8” activity was quantitated as shown in Fig 5. Data shown are representative of three different experiments.
(0.51 MB EPS)

Figure S4 pMHC I expression requires new protein synthesis. (A) Kb+ HEK293 cells expressing EBNAα or ΔGAα were cultured for 24 h in the presence of doxycycline. Cells were briefly treated with mild-acid and used as APCs for B3Z T cells or analyzed for (B) GFP and Kb expression by flow cytometry using PE-conjugated anti-Kb Mab 5F1. (C) Amounts of SHL8 peptide in cells extracted at the indicated time periods after the acid wash. Data show mean ± SD of duplicates and are representative of three different experiments.
(1.09 MB EPS)
